# QuickStats

**Published:** 2015-05-22

**Authors:** FROM THE NATIONAL CENTER FOR HEALTH STATISTICS

**Figure:**
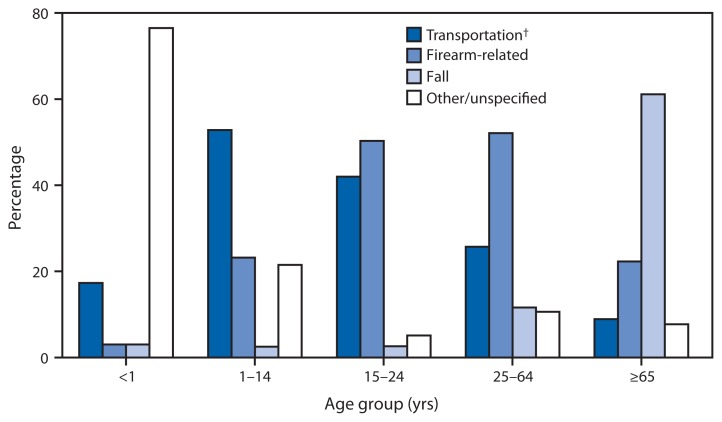
Percentage of Traumatic Brain Injury (TBI)–Related Deaths,* by Underlying Cause and Age Group — United States, 2013 * TBI-related deaths were identified using the *International Classification of Diseases, Tenth Revision* underlying cause of death codes of *U01–*U03, V01–Y36, Y85–Y87, or Y89 with a multiple cause of death code of S01.0–S01.9, S02.0, S02.1, S02.3, S02.7–S02.9, S04.0, S06.0–S06.9, S07.0, S07.1, S07.8, S07.9, S09.7–S09.9, T01.0, T02.0, T04.0, T06.0, T90.1, T90.2, T90.4, T90.5, T90.8, or T90.9, for a total of 54,185 deaths in 2013 for all ages. ^†^ Transportation includes all modes, such as motor vehicle, motorcycle, pedal cycle, pedestrian, other land transport, railway, watercraft, and aircraft.

The causes of injury that result in TBI-related deaths vary by age group. In 2013, 77% of the TBI-related deaths among infants aged <1 year were from causes other than transportation, firearms, or falls, and primarily resulted from assault and maltreatment. Transportation accounted for 53% of the TBI-related deaths among children aged 1–14 years. Firearm-related injuries accounted for 50% and 52% of the TBI-related deaths for persons aged 15–24 and 25–64 years, respectively. Most of the firearm-related TBI deaths in these two age groups were suicides (62% and 83%, respectively). The majority (61%) of TBI-related deaths for those aged ≥65 years resulted from falls.

**Source:** National Vital Statistics System mortality data. Available at http://www.cdc.gov/nchs/deaths.htm. Additional information on TBI available at http://www.cdc.gov/traumaticbraininjury/.

**Reported by:** Holly Hedegaard, MD, HHedegaard@cdc.gov, 301-458-4460.

